# A Longitudinal Study of the Relationship of Adiponectin with Reproduction in Infertile Women Undergoing IVF/ICSI Treatment, and an Experimental Study in Human Granulosa Cells

**DOI:** 10.3390/life13040994

**Published:** 2023-04-12

**Authors:** Lixian Qin, Chantacha Sitticharoon, Somsin Petyim, Issarawan Keadkraichaiwat, Rungnapa Sririwichitchai, Pailin Maikaew, Malika Churintaraphan

**Affiliations:** 1 Department of Physiology, Faculty of Medicine Siriraj Hospital, Mahidol University, 2 Wanglang Rd., Siriraj, Bangkoknoi, Bangkok 10700, Thailand; 2 Department of Obstetrics and Gynecology, Faculty of Medicine Siriraj Hospital, Mahidol University, 2 Wanglang Rd., Siriraj, Bangkoknoi, Bangkok 10700, Thailand

**Keywords:** adiponectin, IVF/ICSI treatment, granulosa cells, aromatase, FSH

## Abstract

This study investigated the roles of adiponectin in IVF treatment during Phase I (the basal stage before gonadotropin administration), Phase II (approximately 8 days after gonadotropin administration), and Phase III (on the ovum pick-up day), as well as the effects of adiponectin on *CYP19A1* and the *FSH receptor* (*FSHR*) mRNA expression in a human granulosa-like tumor cell line (KGN). In human subjects (a longitudinal study, *n* = 30), blood samples were collected in all phases, while follicular fluid (FF) was only collected in Phase III. The participants were classified into successful and unsuccessful groups based on the determination of fetal heartbeats. KGN cells were treated with adiponectin/FSH/IGF-1 (an experimental study, *n* = 3). There was no difference in the adiponectin levels between successful and unsuccessful pregnancies in the FF (Phase III) and in serum (all phases), as well as among the three phases in both groups. Serum FSH (Phase I) was positively associated with serum adiponectin in the unsuccessful group, but it had a negative association in the successful group (all phases). Serum adiponectin and serum FSH (Phase I) were positively correlated in the unsuccessful group, whereas they were negatively correlated (all phases) in the successful group. The serum adiponectin levels (Phase III) were significantly higher than in the FF in unsuccessful pregnancies, but there was no difference in successful pregnancies. FF adiponectin concentrations were negatively correlated with serum LH in successful subjects. In KGN cells, adiponectin had no influence on *CYP19A1* and *FSHR* mRNA expression. High adiponectin levels in serum compared to FF (Phase III) in unsuccessful subjects might negatively impact IVF treatment.

## 1. Introduction

The global health problem of infertility is clinically described as the failure to become pregnant after 12 months of frequent unprotected intercourse [1]. Reproductive health is well known to be tightly regulated by numerous adipokines that are secreted by adipose tissue [2]. One of the most abundant adipokines is adiponectin, and the effects of adiponectin on reproductive regulation have been investigated intensively [3].

Adiponectin is a 244 amino acid glycoprotein which circulates in blood as a full-length structure or homomultimers with low (LMW), medium (MMW), and high (HMW) molecular weights [2]. Adiponectin is synthesized in adipocytes; surprisingly, serum adiponectin levels are negatively correlated with adiposity [4] because elevated pro-inflammatory cytokines, such as tumor necrosis factor-α and interleukin-1β [5], suppress adiponectin secretion in the obese state [6]. Adiponectin affects its target tissues through adiponectin receptor 1 (AdipoR1) and 2 (AdipoR2) [3]. Adiponectin receptors are expressed in the tissues of the hypothalamic–pituitary–gonadal axis, which is the predominant regulator for the reproductive system [3]. In the hypothalamus, adiponectin treatment on gonadotropin-releasing hormone (GnRH) neurons, which express AdipoR1 and AdipoR2, inhibit GnRH neuronal activity and secretion in vitro [7,8]. In the pituitary, both AdipoR1 and AdipoR2 were detected in gonadotropes that synthesize gonadotropins, including the follicle-stimulating hormone (FSH) and luteinizing hormone (LH) [2]. A previous study showed that adiponectin attenuated LH secretion in LβT2 gonadotropes and in mice, but it had no effect on FSH secretion in mice [9]. These results suggest that adiponectin might exert inhibitory effects on the GnRH and LH. Furthermore, the presence of AdipoR1 and AdipoR2 has been detected in peripheral reproductive tissues, including the endometrium and ovarian theca and granulosa cells [2], indicating that adiponectin may have a direct effect on these organs.

One of the major functions of peripheral reproductive tissues is to produce sex steroid hormones, which refers to steroidogenesis. Steroid hormones are synthesized from cholesterol, which is converted to androgen by the activation of signaling pathways after the binding of the LH to its receptor on theca cells [10]. Androgen is then translocated into granulosa cells undergoing aromatization to estrogen, which is achieved via the activation of signaling cascades triggered by the FSH binding to the FSH receptor (FSHR) [10]. Adiponectin with insulin-like growth factor-1 (IGF-1) significantly elevated the aromatase protein expression and estradiol (E2) secretion in human granulosa cells when compared to IGF-1 alone; however, adiponectin could not enhance the FSH-stimulated aromatase protein expression and E2 secretion [11].

Furthermore, adiponectin has been shown to have a positive association with levels of anti-Müllerian hormone (AMH) [12], which is secreted from follicles in the preantral and antral stages, and has been recognized as a key indicator of ovarian reserve [13]. Serum AMH levels are strongly correlated with the positive outcomes of in vitro fertilization (IVF) treatment, which is an effective treatment for infertility [14]. A previous study investigated adiponectin levels in IVF treatment and found that basal serum adiponectin levels were significantly higher in successful pregnancies than unsuccessful pregnancies [15]. However, the follicular fluid (FF) adiponectin levels of successful pregnancies were not different from those of unsuccessful pregnancies [16]. Therefore, the roles of adiponectin in IVF treatment and steroidogenesis must be investigated further.

The present study evaluated the effects of adiponectin among various phases of IVF/intracytoplasmic sperm injection (ICSI) treatment and on steroidogenesis in human granulosa cells. Our objectives were to: (1) compare serum adiponectin concentrations among various phases of IVF/ICSI treatment, consisting of the basal stage before gonadotropin administration (Phase I), approximately 8 days after gonadotropin administration (Phase II), and on the ovum pick-up (OPU) day (Phase III), and the FF adiponectin levels were measured at Phase III and compared between the successful and unsuccessful groups; (2) compare serum adiponectin concentrations among various phases of IVF/ICSI treatment in the successful and unsuccessful subjects; (3) compare adiponectin concentrations between blood and FF in the successful and unsuccessful subjects; and (4) determine the correlations of concentrations of adiponectin in blood and FF with concentrations of other hormones. In human granulosa-like tumor cells (KGN), the direct effects of adiponectin on the mRNA expressions of *CYP19A1* (aromatase) and *FSHR* were investigated.

## 2. Materials and Methods

### 2.1. Participants

The study in humans was a longitudinal study and was conducted at the Faculty of Medicine Siriraj Hospital, Mahidol University, Thailand. Infertile women with the cause of infertility and/or their male partners with oligospermia undergoing IVF/ICSI treatment were enrolled from April 2018 to May 2019. The inclusion criteria of this study comprised women aged 25–40 years with a normal body mass index (BMI) (18.5–24.9 kg/m^2^), regular menstrual cycles lasting 28–31 days, and both intact ovaries. The exclusion criteria were patients with a history of hormonal contraceptive utilization within two months, endocrine diseases, moderate/severe endometriosis, polycystic ovarian syndrome (PCOS), and a poor response to IVF treatment. Participants with obesity (BMI < 25 kg/m^2^ based on the Asians BMI classification) [17] and PCOS were excluded to limit the confounding effects of obesity or abnormal hormonal levels found in PCOS on the success of IVF treatment [3]. All the participants (*n* = 30) signed the informed consent forms before the initiation of this study. Among them, 10 patients had a successful clinical pregnancy (successful group) and 19 patients had an unsuccessful clinical pregnancy (unsuccessful group). Additionally, a patient who had an ectopic pregnancy was recruited for the purpose of correlation analysis.

### 2.2. IVF/ICSI Treatment Procedure

The treatment processes were based on the standard IVF/ICSI treatment procedures. During the first two or three days of the menstrual cycle (Phase I), a once-daily subcutaneous injection of recombinant FSH (rFSH; GONAL-f, Merck Serono, Darmstadt, Germany) was used to stimulate the development of multiple follicles for 11 days. Prior to injection, the blood levels of the FSH, LH, AMH, progesterone (P4), E2, and adiponectin were measured. Endovaginal sonography (SSD-3500SX, Aloka, Tokyo, Japan) was performed to examine the uterus and ovary pathology. After 6 days of rFSH injection, a once-daily subcutaneous GnRH antagonist administration (0.25 mg of Cetrotide, Merck Serono, Darmstadt, Germany) was initiated for the prevention of premature LH surge. Approximately 8 days after ovarian stimulation (Phase II), the blood concentrations of the FSH, LH, P4, E2, and adiponectin were measured. The development of each follicle was evaluated using transvaginal ultrasound. On approximately day 12 of the menstrual cycle, human chorionic gonadotropin (hCG) (250 mg/0.5 mL of Ovidrel, Merck Serono, Darmstadt, Germany) was injected subcutaneously to trigger oocyte maturation.

After 36 h of hCG injection, approximately on day 14 of the menstrual cycle, OPU (Phase III) was performed using endovaginal ultrasound to collect oocytes and FF. The blood levels of the FSH, LH, P4, E2, and adiponectin were measured just before the OPU. After the removal of oocytes, FF was collected to analyze adiponectin levels. After oocyte retrieval, ICSI was performed to fertilize the metaphase II oocytes. Three days after OPU, one to two qualified embryo(s) was/were transferred into the uterine cavity. Approximately 15 days after the embryo transfer, blood hCG was measured to test for biochemical pregnancy. On approximately day 42 of the menstrual cycle, in the case of biochemical pregnancy being achieved, clinical pregnancy was verified by the detection of the fetal heartbeat through transabdominal ultrasound.

### 2.3. Hormonal Measurement

Electrochemiluminescence immunoassay kits (COBAS^®^, Roche Diagnostics GmbH, Mannheim, Germany) were used for measuring the serum FSH, LH, AMH, P4, and E2 at the central laboratory of the Department of Clinical Pathology, Faculty of Medicine, Siriraj Hospital. In accordance with the guidelines provided by the manufacturer, the detection ranges of the FSH, LH, AMH, P4, and E2 were 0.1–200 mIU/mL, 0.1–200 mIU/mL, 0.01–23 ng/mL, 0.05–60 ng/mL, and 5–3000 pg/mL, respectively.

The serum and FF levels of total adiponectin were detected using enzyme-linked immunosorbent assay kits (Phoenix Pharmaceuticals, Burlingame, CA, USA) in accordance with the manufacturer’s instructions, with a detection range of 0–100 ng/mL. The intra-assay variation in the experiments was 3.37%.

### 2.4. The KGN Cell Treatments

The study in KGN cells was an experimental study, which were commercially acquired from the RIKEN Bioresource center (Tsukuba, Japan). The KGN cells were cultured in a humidified incubator at 37 °C with 5% CO_2_, using a 1:1 mixture of Dulbecco’s modified Eagle’s medium/nutrient Ham’s mixture F-12 (DMEM/F-12) medium (Gibco, Carlsbad, CA, USA) supplemented with 1% penicillin–streptomycin (Sigma-Aldrich, St. Louis, MO, USA) and 10% fetal bovine serum (FBS, Hyclone, South Logan, UT, USA). After the fourth passage, the KGN cells were incubated with serum-free DMEM/F-12 medium as the control; 10^−8^ M FSH (Abcam, Cambridge, UK); 10^−8^ M insulin-like growth factor-1 (IGF-1) (Sigma-Aldrich, St. Louis, MO, USA); 10^−8^ M FSH together with 10^−8^ M IGF-1 (FSH+IGF-1); 300 nM of globular adiponectin (Adipo) (Merck KGaA, Darmstadt, Germany); and 300 nM of adiponectin together with 10^−8^ M FSH and 10^−8^ M IGF-1 (Adipo+FSH+IGF-1). The doses of adiponectin, FSH, and IGF-1 were chosen according to a previous study [11]. The experiments, including the control, were performed in triplicate. After incubation for 48 h, the cell extracts and medium were used for further analysis.

### 2.5. RNA Extraction and Real-Time Polymerase Chain Reaction (RT-PCR)

RNA extraction from KGN cells was performed using the TRIzol^®^ Reagent (Invitrogen, Carlsbad, CA, USA) to determine genes of interest, including *FSHR* and *CYP19A1*, and a reference gene, which was *hypoxanthine phosphoribosyltransferase 1* (*HPRT1*) [18].

For each sample, 500 ng of extracted RNA was converted to cDNA through reverse transcription using the iScript cDNA^TM^ synthesis kit (Bio-Rad, Hercules, CA, USA). In accordance with the manufacturer’s instructions, the cDNA was amplified as follows: 5 min at 25 °C, 30 min at 42 °C, and 5 min at 85 °C using the MasterCycler gradient (Eppendorf, Hamburg, Germany), which then was kept at −20 °C until RT-PCR analysis. The primer sequences of the *FSHR*, *CYP19A1*, and *HPRT1* genes were designed by the authors and published previously [19] as follows: *FSHR* (240 base pairs): forward—5′ AGGAATGCCATTGAACTGAGGT 3′, reverse—5′ CAGATATTGAAGGTTGGGAAGGT 3′; *CYP19A1* (267 base pairs): forward—5′ AGCAAGTCCTCAAGTATGTTCCAC 3′, reverse—5′ GTCCACATAGCCCGATTCATT 3′; and *HPRT1* (156 base pairs): forward—5′ CCTGGCGTCGTGATTAGTGA 3′, reverse—5′ CCATCTCCTTCACATCTCG 3′.

In accordance with the instructions provided by the manufacturer, the RT-PCR procedures were performed using the qPCR green master mix LRox, 2X kit (biotechrabbit, Berlin, Germany) as follows: the initiation of the Taq DNA polymerase at 50 °C for 2 min; the activation of the Taq DNA polymerase at 95 °C for 3 min; 40 cycles of PCR amplification including denaturation at 95 °C for 15 s, annealing at 60 °C for *CYP19A1* and *HPRT1* and at 55 °C for *FSHR* for 30 s, and extension at 65 °C for 30 s; and the melting process involving denaturation at 95 °C for 1 min, renaturation at 55 °C for 30 s, and melting at 95 °C for 30 s using the CFX96 real-time PCR detection system (Bio-Rad, Hercules, CA, USA). Each experiment was duplicated. For the negative control, DNase- and RNase-free distilled water was utilized to substitute the cDNA template (no-template control). Then, the 2^−ΔCt^ method was performed to determine the *FSHR* and *CYP19A1* gene expressions.

### 2.6. Statistical Analysis

The Statistical Package for the Social Sciences (version 18.0, IBMS SPSS, Armonk, NY, USA) was used to analyze the statistics in this study. For humans, information regarding age, body weight, BMI, the duration of the menstrual cycle, the duration of ovarian stimulation, serum FSH, and serum LH in the three phases of the IVF/ICSI treatment were presented as the 25th percentile, median, and 75th percentile. The mean values of these parameters and the differences between successful and unsuccessful pregnancies were reported in a previously published paper [19]. Other data are shown as the mean ± standard deviation (SD). The normal distribution of data was assessed by the Kolmogorov–Smirnov test. An independent Student’s *t*-test was applied to make comparisons between the successful and unsuccessful groups, while a paired *t*-test was used for comparing serum and FF in successful and unsuccessful subjects. To compare between the different stages of IVF/ICSI treatment in both successful and unsuccessful groups, as well as the groups of KGN cells, a one-way analysis of variance was carried out, followed by Fisher’s least significant difference post hoc test where appropriate. Where the data were not normally distributed, non-parametric tests were performed. To conduct a correlation analysis, normally distributed data were assessed using Pearson’s product–moment correlation coefficient, while non-normally distributed data were evaluated using the Spearman’s rank correlation coefficient. The level of statistical significance was defined as a *p*-value less than 0.05.

## 3. Results

### 3.1. Demographic and Clinical Data of Subjects

Demographic data consisting of age, body weight, and BMI, as well as clinical data containing the duration of the menstrual cycle, the duration of ovarian stimulation, and the serum levels of the FSH and LH in Phases I–III in both the successful and unsuccessful groups are shown in Table 1 as the 25th percentile, median, and 75th percentile.

### 3.2. Comparisons of Serum Adiponectin Levels between the Successful and Unsuccessful Pregnancies in Various Phases, as Well as FF Adiponectin Levels in Phase III of the IVF/ICSI Treatment

Comparisons of serum adiponectin levels between the successful and unsuccessful pregnancies in various phases, as well as the FF adiponectin levels in Phase III of IVF/ICSI treatment, are shown in Table 2. Serum adiponectin levels in Phases I–III, as well as the FF adiponectin levels in Phase III of IVF/ICSI treatment, were comparable between successful and unsuccessful subjects (Table 2).

### 3.3. Comparisons of Serum Adiponectin Levels among Various Phases, as Well as between Serum and FF in Total, and Successful and Unsuccessful Pregnancies in Phase III of the IVF/ICSI Treatment

**Figure 1:**
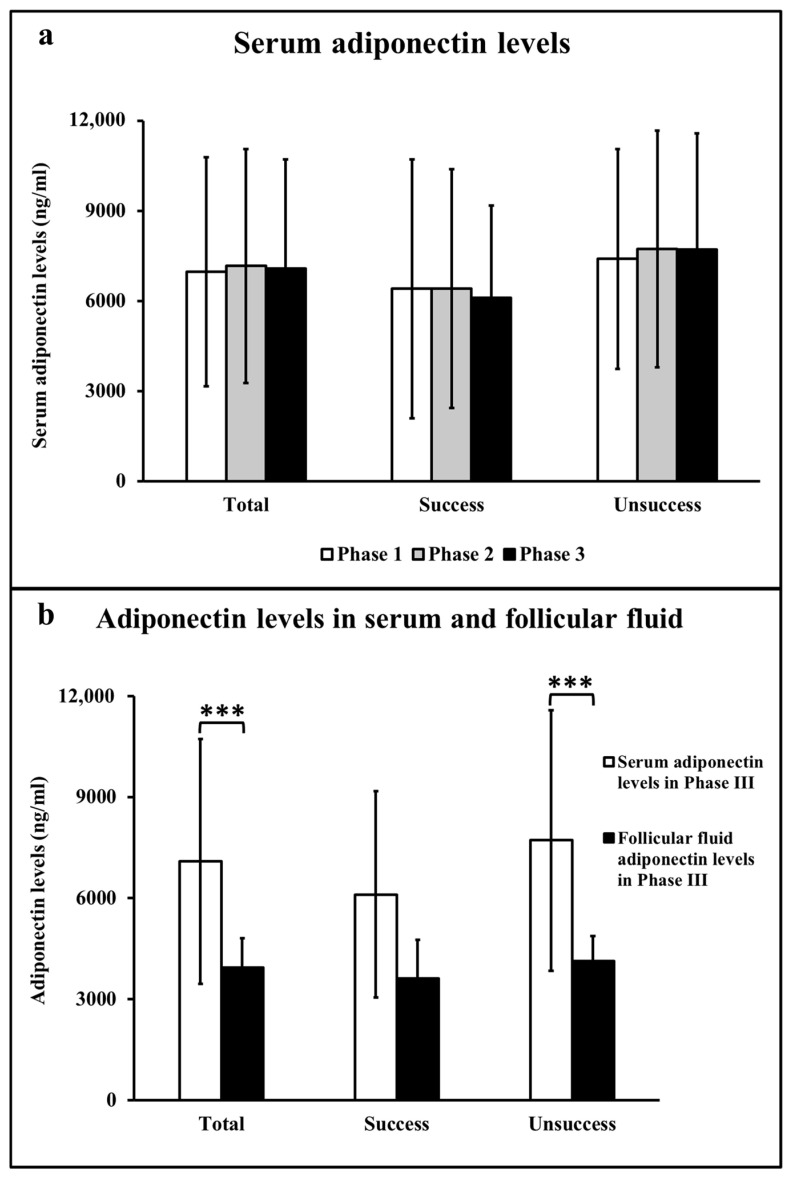
Comparisons of serum adiponectin levels among various phases, as well as between serum and follicular fluid (FF) in total (*n* = 29), successful (*n* = 10), and unsuccessful groups (*n* = 19) in Phase III of the in vitro fertilization (IVF)/intracytoplasmic sperm injection (ICSI) treatment. (**a**) Comparisons of serum adiponectin levels among the various phases. (**b**) Comparisons of adiponectin levels between serum and FF in Phase III. Phase I: basal stage before gonadotropin administration (the early follicular phase); Phase II: approximately 8 days after gonadotropin administration (the late follicular phase); and Phase III: on the ovum pick-up day (the ovulatory phase). Values are reported as mean ± SD, *** *p* < 0.001.

Comparisons of the serum adiponectin levels among the various phases of IVF/ICSI treatment are shown in Figure 1a. The serum adiponectin levels were not different among all phases (Phases I–III) of IVF/ICSI treatment in total, or between successful and unsuccessful pregnancies (Figure 1a), even after being adjusted for the basal FSH or basal AMH.

Comparisons of adiponectin levels between serum and FF in total, and in successful and unsuccessful pregnancies during Phase III are presented in Figure 1b. The adiponectin levels in serum were statistically greater than those in FF in total and in the unsuccessful groups, while they were not different in the successful groups (Figure 1b).

### 3.4. Correlations of Serum and FF Adiponectin Levels with hormonal Levels in IVF/ICSI Treatment

The correlations of serum and FF adiponectin levels with hormonal levels during IVF/ICSI treatment are shown in Table 3.

The levels of serum adiponectin in Phase I were positively correlated with the levels of serum adiponectin in Phase II in all (R = 0.977, *p* < 0.001), successful (R = 0.964, *p* < 0.001), and unsuccessful subjects (R = 0.982, *p* < 0.001), and in Phase III in all (R = 0.935, *p* < 0.001) and unsuccessful subjects (R = 0.961, *p* < 0.001), with a trend of a positive correlation in successful subjects (R = 0.667, *p* = 0.050) (Table 3).

The levels of serum FSH in Phase I were positively correlated with the levels of serum adiponectin in Phase I (R = 0.528, *p* = 0.020), Phase II (R = 0.491, *p* = 0.038), and Phase III (R = 0.481, *p* = 0.037) in unsuccessful subjects and in Phase III (R = 0.392, *p* = 0.035) in all subjects, but were negatively correlated with the levels of serum adiponectin in Phase I (R = −0.648, *p* = 0.043) and Phase II (R = −0.648, *p* = 0.043) in successful subjects (Table 3).

The FF adiponectin levels showed trends of a positive correlation with serum adiponectin in Phase I (R = 0.386, *p* = 0.051), Phase II (R = 0.395, *p* = 0.051), and Phase III (R = 0.345, *p* = 0.084) in all subjects and in Phase II (R = 0.482, *p* = 0.059) and Phase III (R = 0.480, *p* = 0.051) in unsuccessful subjects, but they had a significantly negative correlation with the serum LH levels in Phase III (R = −0.714, *p* = 0.047) in successful subjects (Table 3).

The serum LH in Phase II tended to have a negative correlation with the serum adiponectin in Phase II in unsuccessful subjects (R = −0.419, *p* = 0.074).

### 3.5. Summary of Results for Adiponectin in the IVF/ICSI Treatment

**Figure 2:**
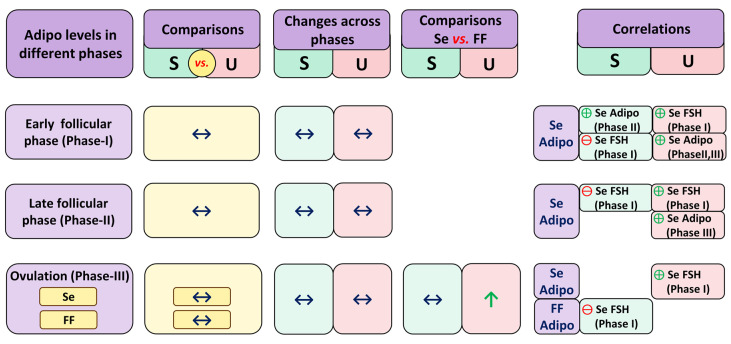
Summary of results for adiponectin in the in vitro fertilization (IVF)/intracytoplasmic sperm injection (ICSI) treatment. S: successful; U: unsuccessful; Se: serum; FF: follicular fluid; Adipo: adiponectin; FSH: follicle-stimulating hormone; LH: luteinizing hormone; Phase I: basal stage before gonadotropin administration (the early follicular phase); Phase II: approximately 8 days after gonadotropin administration (the late follicular phase); and Phase III: on the ovum pick-up day (the ovulatory phase); 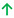: increased, 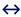: comparable, 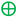: positive correlation, 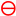: negative correlation.

The summary of the results for adiponectin in IVF/ICSI treatment is shown in Figure 2.

### 3.6. Effects of Adiponectin on CYP19A1 and FSHR mRNA Expression in KGN Cells

**Figure 3:**
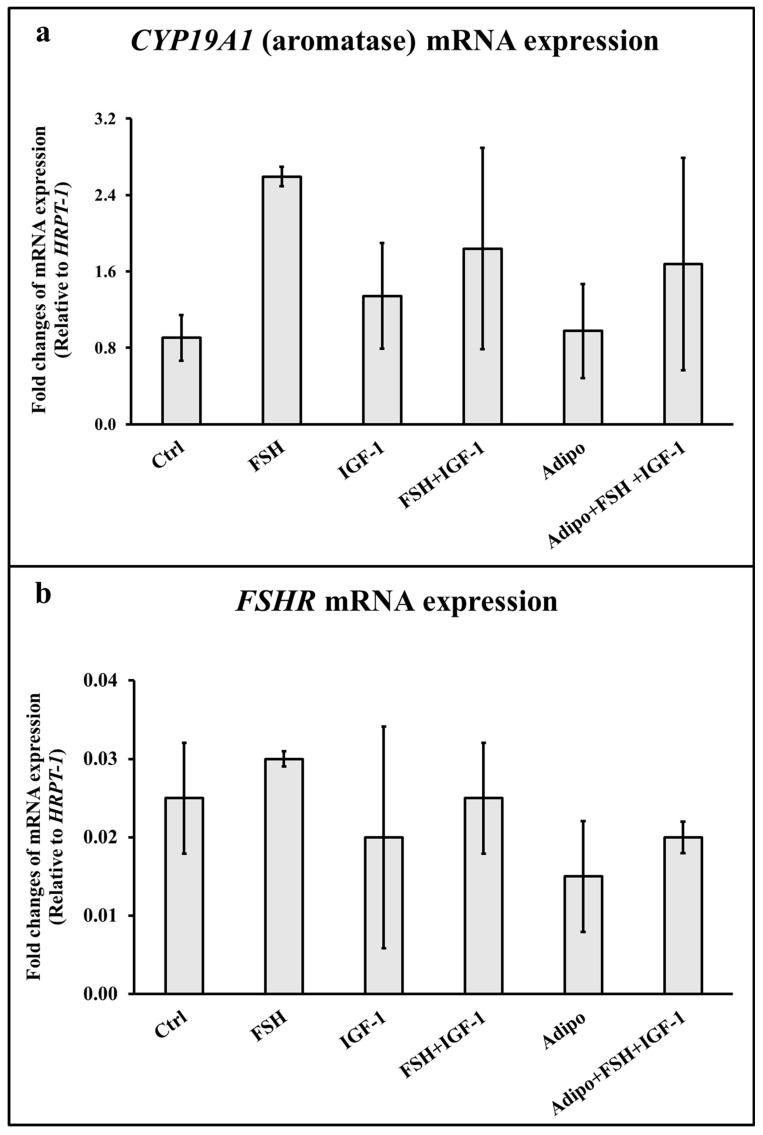
Effects of adiponectin on the *CYP19A1* (aromatase) and *follicle-stimulating hormone receptor* (*FSHR*) mRNA expression in KGN cells (*n* = 3). (**a**) *CYP19A1* mRNA expression; (**b**) *FSHR* mRNA expression. Ctrl: control; FSH: 10^−8^ M follicle-stimulating hormone; IGF-1: 10^−8^ M insulin-like growth factor-1; Adipo: 300 nM adiponectin. Values are presented as mean ± SD.

The effects of adiponectin on the *CYP19A1* and *FSHR* mRNA expression in KGN cells are shown in Figure 3. Treatments with the FSH, IGF-1, FSH+IGF-1, Adipo, and Adipo+FSH+IGF-1 had no effects on the *CYP19A1* (Figure 3a) and *FSHR* (Figure 3b) mRNA expression compared to the control group.

## 4. Discussion

The present study determined the levels of serum adiponectin in three phases and FF adiponectin in Phase III of IVF/ICSI treatment. In addition, this study also investigated the direct impacts of adiponectin on ovarian steroidogenesis. To the best of our knowledge, this is the first investigation to compare serum adiponectin levels across the three phases of the IVF/ICSI treatment in successful and unsuccessful subjects and to explore the associations of serum adiponectin and FF adiponectin levels with hormonal levels in successful and unsuccessful subjects.

This study revealed that serum adiponectin levels were not different between the successful and unsuccessful subjects in all three phases of the IVF/ICSI treatment and they did not change across the various phases of IVF/ICSI treatment in either group. These findings were in accordance with previous publications revealing that adiponectin-null mice were not related to a diminished reproductive ability [20,21,22]. We postulated that adiponectin might not exert beneficial effects on the outcome of IVF/ICSI treatment. Furthermore, in the KGN cell experiments, adiponectin treatment did not impact the *FSHR* and *CYP19A1* mRNA expression, which parallels a previous study showing that adiponectin together with FSH treatment did not enhance aromatase protein expression or estrogen secretion when compared to the FSH alone [11]. Considering human and KGN cell studies, adiponectin might not play an important role in IVF/ICSI treatment.

Remarkably, the serum levels of adiponectin in Phase III were significantly greater than the FF levels in unsuccessful subjects, while the levels were comparable in successful subjects. Our findings in unsuccessful subjects were in agreement with a previous publication revealing that adiponectin levels in FF were lower than in serum [23], suggesting that adiponectin might not be primarily secreted from follicular cells. Our hypothesis was supported by a previous study showing that adiponectin was mainly and abundantly secreted by adipose tissue [24]. Adiponectin protein expression was not detected in primary granulosa cells from patients who had undergone IVF [11]. Furthermore, a previous study found that the major isoform of adiponectin was of an LMW in FF but an HMW in serum, and the LMW isoform was transferred faster than the HMW one [23], suggesting that FF adiponectin might be passively diffused from the blood.

The serum-to-FF ratio of adiponectin was 1.68 in the successful group and 1.87 in the unsuccessful group. Furthermore, serum adiponectin levels in Phase II had a trend of a negative correlation with the serum LH in Phase II in unsuccessful subjects. The serum adiponectin levels in Phase III had a significantly positive correlation with the serum FSH levels in Phase I (basal FSH) in total and unsuccessful pregnancies, but they had no significant correlation in successful pregnancies. Additionally, a negative correlation between FF adiponectin in Phase III and the serum LH in Phase III was observed only in the successful group. These results suggest that a high ratio of serum-to-FF adiponectin in Phase III and high FF adiponectin levels might be related to unsuccessful pregnancies. Earlier studies have shown that a high basal FSH associated with low levels of inhibin B [25,26,27] leads to a decreased inhibitory effect on the FSH [28], and the poor responsiveness of ovarian cells to the FSH due to a reduced *FSHR* expression in granulosa cells and/or the aging process [29,30], which is related to a diminished ovarian reserve [31] and failed consequences of IVF treatment [32,33,34,35]. In addition, high levels of serum adiponectin in Phase II could be associated with low levels of serum LH in Phase II. The LH is a key coordinator, along with the FSH, to stimulate healthy follicular development [36] and it exerts the conversion of P4 to androgen, which is further aromatized to estrogen, with synergistic effects on the FSH to enhance follicular growth in mice [37,38]. Furthermore, decreased levels of FF adiponectin could be associated with elevated levels of serum LH in Phase III. The sharp rise of the LH in the preovulatory phase (immediately prior to Phase III) is crucial for triggering ovulation [39]. As a result, we postulated that a positive correlation between serum adiponectin in Phase III and a high basal FSH denoted the trend of a negative correlation between serum adiponectin and the LH in Phase II, as well as a negative correlation between FF adiponectin and serum LH levels in Phase III, indicating that high levels of serum/FF adiponectin in Phase II/III might negatively impact IVF/ICSI outcomes.

We hypothesized that the higher serum levels of adiponectin in Phase II/III could suppress GnRH neuronal activity, leading to attenuated GnRH secretion; therefore, such levels would likely attenuate the LH’s actions. However, the ability of adiponectin to cross the blood–brain barrier (BBB) to suppress the release of GnRH has not been fully determined. Previous studies have reported that COOH-terminal FLAG tag-labeled adiponectin was detected in cerebrospinal fluid (CSF) in rats [40] after an intravenous injection and adiponectin administration peripherally enhanced its levels in CSF in mice [41], indicating that adiponectin could cross the BBB. Moreover, adiponectin was detected in human CSF [40] with abundant trimer, fewer hexamer, and fewer multimer isoforms [42]. However, another article reported that adiponectin was not present in human CSF, and radiolabeled glycosylated or non-glycosylated adiponectin protein was undetected in mice brain after an intravenous injection [43], indicating that adiponectin could not cross the BBB. Based on these results, it is unclear whether adiponectin can cross the BBB to exert an inhibitory effect on GnRH secretion. However, adiponectin was shown to directly inhibit LH secretion from gonadotrope cells to attenuate any LH surge [3,9,44]. Taken together, we propose that high adiponectin levels in blood circulation might be associated with high basal FSH and FF adiponectin levels, which could exert adverse effects on IVF/ICSI treatment that are probably related to decreased LH concentrations.

## 5. Limitations

First, FF adiponectin levels could not be measured in Phases I and II of the IVF/ICSI treatment due to ethical limitations. Second, the human study had a small sample size, particularly in the successful group, which resulted in fewer statistically significant observations when comparing successful and unsuccessful pregnancies, as well as in correlation analysis. Third, only *FSHR* and *CYP19A1* mRNA expression were reported in the KGN cell experiment, which is insufficient to confirm the role of adiponectin. Further studies providing protein levels of FSHR and CYP19A1 are important to confirm the findings from the mRNA data.

## 6. Conclusions

Our results revealed that serum adiponectin levels in Phase III were significantly elevated compared to the serum adiponectin levels in the FF in unsuccessful pregnancies, while they were not significantly different in successful pregnancies. In addition, the serum adiponectin levels in Phase III had a significantly positive correlation with the basal FSH levels in the unsuccessful group. Furthermore, in Phase III, the FF adiponectin levels were negatively correlated with the serum LH levels in the successful group. High serum adiponectin levels compared to FF in Phase III in unsuccessful subjects could potentially have a negative impact on IVF treatment. In the KGN cell experiments, adiponectin did not have any effect on *CYP19A1* and *FSHR* mRNA expression. 

## Figures and Tables

**Table 1:** Demographic and clinical data of subjects.

Parameters	Success (*n* = 10)			Unsuccess (*n* = 19)		
	P25	Median	P75	P25	Median	P75
Age (years)	33.25	35.5	36.50	34.00	36.00	37.00
Body weight (kg)	50.48	57.00	60.00	47.10	50.00	57.70
BMI (kg/m^2^)	19.13	21.21	23.46	19.22	20.34	21.67
Duration of the menstrual cycle (days)	28.75	29.00	34.00	27.00	28.00	29.00
Duration of ovarian stimulation (days)	9.50	11.00	12.00	9.00	10.00	10.00
Serum FSH at Phase I (mIU/mL)	5.24	6.35	7.72	6.97	7.89	11.22
Serum FSH at Phase II (mIU/mL)	10.11	14.05	16.89	13.58	18.49	21.58
Serum FSH at Phase III (mIU/mL)	9.03	9.89	10.68	6.96	10.05	12.87
Serum LH at Phase I (mIU/mL)	3.57	4.55	6.30	4.56	5.15	7.55
Serum LH at Phase II (mIU/mL)	1.17	1.76	4.01	1.03	1.89	2.83
Serum LH at Phase III (mIU/mL)	0.77	3.21	3.95	0.37	0.56	3.12

P25: 25th percentile; P75: 75th percentile; BMI: body mass index; FSH: follicle-stimulating hormone; LH: luteinizing hormone; Phase I: basal stage before gonadotropin administration (the early follicular phase); Phase II: approximately 8 days after gonadotropin administration (the late follicular phase); and Phase III: on the ovum pick-up day (the ovulatory phase).

**Table 2:** Comparisons of serum adiponectin levels between successful and unsuccessful pregnancies in various phases, as well as FF adiponectin levels in Phase III of the IVF/ICSI treatment.

Parameters	Success (*n* = 10)	Unsuccess (*n* = 19)	*p*-Value
Serum adiponectin (Phase I) (ng/mL)	6402.98 ± 4304.51	7401.69 ± 3658.97	0.516
Serum adiponectin (Phase II) (ng/mL)	6410.69 ± 3980.85	7725.20 ± 3939.70	0.407
Serum adiponectin (Phase III) (ng/mL)	6109.20 ± 3060.51	7708.91 ± 3860.96	0.287
FF adiponectin (ng/mL)	3619.07 ± 1150.10	4128.02 ± 745.42	0.195

FF: follicular fluid; Phase I: basal stage before gonadotropin administration (the early follicular phase); Phase II: approximately 8 days after gonadotropin administration (the late follicular phase); and Phase III: on the ovum pick-up day (the ovulatory phase). Values are reported as mean±SD.

**Table 3:** The correlations between serum adiponectin, FF adiponectin, and hormonal levels in the various phases of the IVF/ICSI treatment.

	Parameters	All (*n* = 30)		Success (*n* = 10)		Unsuccess (*n* = 19)	
		R	*p*	R	*p*	R	*p*
Serum adiponectin (Phase I)	Serum adiponectin (Phase II)	0.977	<0.001 ***	0.964	<0.001 ***	0.982	<0.001 ***
	Serum adiponectin (Phase III)	0.935	<0.001 ***	0.667	0.050	0.961	<0.001 ***
Serum FSH (Phase I)	Serum adiponectin (Phase I)	0.320	0.084	−0.648	0.043 *	0.528	0.020 *
	Serum adiponectin (Phase II)	0.336	0.075	−0.648	0.043 *	0.491	0.038 *
	Serum adiponectin (Phase III)	0.392	0.035 *	−0.567	0.112	0.481	0.037 *
FF adiponectin	Serum adiponectin (Phase I)	0.386	0.051	0.429	0.289	0.424	0.090
	Serum adiponectin (Phase II)	0.395	0.051	0.238	0.570	0.482	0.059
	Serum adiponectin (Phase III)	0.345	0.084	−0.095	0.823	0.480	0.051
	Serum LH (Phase III)	0.082	0.691	−0.714	0.047 *	0.346	0.173

FSH: follicle-stimulating hormone; LH: luteinizing hormone; FF: follicular fluid; Phase I: basal stage before gonadotropin administration (the early follicular phase); Phase II: approximately 8 days after gonadotropin administration (the late follicular phase); and Phase III: on the ovum pick-up day (the ovulatory phase), * *p* < 0.05, *** *p* < 0.001.

## Data Availability

The data presented in this study are available on request from the corresponding author. The data are not publicly available due to restrictions i.e. their containing information that could compromise the privacy of research participants.
